# Psychopathic traits modulate brain responses to drug cues in incarcerated offenders

**DOI:** 10.3389/fnhum.2014.00087

**Published:** 2014-02-24

**Authors:** Lora M. Cope, Gina M. Vincent, Justin L. Jobelius, Prashanth K. Nyalakanti, Vince D. Calhoun, Kent A. Kiehl

**Affiliations:** ^1^Department of Psychology, University of New MexicoAlbuquerque, NM, USA; ^2^The Mind Research NetworkAlbuquerque, NM, USA; ^3^Department of Psychiatry, University of Massachusetts Medical SchoolWorcester, MA, USA; ^4^Department of Electrical and Computer Engineering, University of New MexicoAlbuquerque, NM, USA

**Keywords:** psychopathic traits, fMRI, drug craving, substance dependence, incarcerated offenders, paralimbic system

## Abstract

Recent neuroscientific evidence indicates that psychopathy is associated with abnormal function and structure in limbic and paralimbic areas. Psychopathy and substance use disorders are highly comorbid, but clinical experience suggests that psychopaths abuse drugs for different reasons than non-psychopaths, and that psychopaths do not typically experience withdrawal and craving upon becoming incarcerated. These neurobiological abnormalities may be related to psychopaths' different motivations for—and symptoms of—drug use. This study examined the modulatory effect of psychopathic traits on the neurobiological craving response to pictorial drug stimuli. Drug-related pictures and neutral pictures were presented and rated by participants while hemodynamic activity was monitored using functional magnetic resonance imaging. These data were collected at two correctional facilities in New Mexico using the Mind Research Network mobile magnetic resonance imaging system. The sample comprised 137 incarcerated adult males and females (93 females) with histories of substance dependence. The outcome of interest was the relation between psychopathy scores (using the Hare Psychopathy Checklist-Revised) and hemodynamic activity associated with viewing drug-related pictures vs. neutral pictures. There was a negative association between psychopathy scores and hemodynamic activity for viewing drug-related cues in the anterior cingulate, posterior cingulate, hippocampus, amygdala, caudate, globus pallidus, and parts of the prefrontal cortex. Psychopathic traits modulate the neurobiological craving response and suggest that individual differences are important for understanding and treating substance abuse.

## Introduction

Substance abuse and psychopathy are two conditions strongly linked to criminal activity. The net annual burden of crime in the United States alone has been estimated to exceed $3.2 trillion (Anderson, 2011; Kiehl and Hoffman, 2011). Individuals with psychopathy are among the most dangerous and chronic offenders, as evidenced by high re-offense rates (Hemphill et al., 1998; Leistico et al., 2008). Moreover, the use of alcohol and drugs greatly increases the likelihood of psychopathic individuals engaging in serious and/or violent criminal activity. Indeed, a large scale study on aggression and offending found that the best predictor of violence was psychopathic traits in conjunction with alcohol and/or drug abuse (Steadman et al., 2000). This is particularly disconcerting given the fact that individuals with psychopathy appear to have a propensity toward substance abuse (Smith and Newman, 1990; Walsh et al., 2007). One potentially effective way to decrease the economic burden of criminal activity in the U.S. would be to design effective substance use treatments for individuals with psychopathy.

Psychopathy is characterized by abnormal affective, interpersonal, and behavioral functioning. Psychopathic traits include emotional deficits such as a profound inability to experience empathy and remorse; behavioral problems such as impulsivity, stimulation-seeking, and instrumental aggression are also noted features (Cleckley, 1976; Hare, 2003). Given this behavioral propensity, it should not be surprising that the comorbidity of psychopathy and substance abuse is high. Relative to non-psychopathic offenders, research has shown that psychopaths are more likely to have a diagnosis of drug abuse or dependence and are more likely to have a polysubstance diagnosis (Smith and Newman, 1990). An interesting paradox may exist, however. Though psychopaths are more likely to be diagnosed with drug use disorders than non-psychopaths, clinical observation suggests that psychopaths are less likely to experience symptoms such as withdrawal and craving when access to drugs is externally limited (e.g., during incarceration) (Cleckley, 1988).

Drug craving is an intense desire or urge to use drugs, and plays a fundamental role in the maintenance of drug use problems. Craving has been associated with both repeated drug use and relapse after a period of abstinence (cf. Ehrman et al., 1998; Weiss, 2005). Cue-elicited craving paradigms, where drug cues are presented and brain activity is recorded using functional imaging techniques, have identified several cortical and subcortical brain regions related to craving^1^: anterior cingulate [Childress et al., 1999 (cocaine); Filbey et al., 2009 (marijuana); Garavan et al., 2000 (cocaine); Heinz et al., 2004 (alcohol); Wang et al., 2011 (heroin); Yin et al., 2012 (methamphetamine)], posterior cingulate (Wang et al., 2011), orbitofrontal cortex [Bonson et al., 2002 (cocaine); Sell et al., 2000 (opioids)], insula [Brody et al., 2002 (nicotine); Myrick et al., 2004 (alcohol); Wang et al., 1999 (cocaine)], ventral and dorsal striatum [David et al., 2005 (nicotine); Garavan et al., 2000; Myrick et al., 2004; Wang et al., 2011] (i.e., nucleus accumbens, caudate, and putamen), thalamus [Franklin et al., 2007 (nicotine); Wang et al., 2011], and amygdala (Childress et al., 1999; Bonson et al., 2002; Franklin et al., 2007). However, despite the strong tendency for individuals with psychopathy to abuse drugs, studies on the neurobiological correlates of craving have not yet examined the potential modulating effect of psychopathic traits.

An examination of these two separate lines of research suggests there is striking overlap between regions implicated in drug craving and regions implicated in psychopathy. For instance, among individuals with psychopathy, reduced amygdala activity has been reported during aversive delay conditioning (Birbaumer et al., 2005), emotional memory (Kiehl et al., 2001), and moral decision-making (Harenski et al., 2010). The orbitofrontal cortex also has received considerable attention due to its role in impulse control. One study identified abnormalities in the orbitofrontal cortex in psychopathic adults during an attention-related task (Veit et al., 2002). Noting these abnormalities, Blair (2008) proposed that amygdala and orbitofrontal cortex/ventromedial prefrontal cortex dysfunction are fundamentally related to the development of psychopathy. Other regions have been shown to be under-reactive, including the anterior cingulate during negative picture viewing (Muller et al., 2003) and aversive conditioning (Veit et al., 2002), the insula during aversive conditioning (Veit et al., 2002; Birbaumer et al., 2005), the ventral striatum and posterior cingulate during an emotional memory task (Kiehl et al., 2001), and the posterior cingulate during emotional moral decision-making (Glenn et al., 2009). Taken together, these results lend support to the paralimbic hypothesis of psychopathy (Kiehl, 2006), which states that limbic (e.g., amygdala, hippocampus) and nearby paralimbic structures (e.g., anterior cingulate, insula, orbitofrontal cortex) are dysfunctional in response to emotional and other salient stimuli.

Notwithstanding years of behavioral, psychophysiological, and now neuroimaging research focusing on reward and punishment processing in psychopathy, the picture is not entirely clear. Despite clinical observation to the contrary, some studies have instead suggested a heightened sensitivity to (monetary) reward with higher psychopathic traits (Buckholtz et al., 2010; Bjork et al., 2012). Another study found a positive correlation between (monetary) gain vs. loss activity in the ventral striatum and psychopathy scores in those scoring 30 or above on the Psychopathy Checklist-Revised (PCL-R) (Hare, 2003; Pujara et al., 2013). Other studies of youth with disruptive behavior disorders (e.g., oppositional defiant disorder, conduct disorder, attention deficit hyperactivity disorder, psychopathic traits) suggest abnormal reward processing (Finger et al., 2008, 2011; White et al., 2013). The present study was undertaken in an effort to clarify how psychopathic traits in adults are related to neural responses to drug cues specifically, given increased drug use but decreased craving and withdrawal among psychopaths.

Studies indicate that individuals with psychopathy start using substances at an earlier age (Corrado et al., 2004) and are more likely to develop polysubstance dependence (Smith and Newman, 1990; Mailloux et al., 1997), but clinical observation points to less craving and withdrawal (Cleckley, 1988). The present study utilized fMRI and a cue-elicited drug craving paradigm to examine the modulatory effect of psychopathic traits on the neurobiological craving response in a sample of 137 incarcerated offenders. Participants viewed drug-related and non-drug pictures, and indicated their level of craving for each picture. Since psychopaths tend not to experience withdrawal and craving and the regions engaged during drug craving overlap considerably with the regions implicated as being deficient in psychopathy, we expected to see reduced craving-related activity in paralimbic, limbic, and subcortical areas among participants with higher scores on the PCL-R. These areas included the anterior cingulate, insula, amygdala, dorsal and ventral striatum, thalamus, and orbitofrontal cortex.

## Materials and methods

### Participants

These data were drawn from a National Institute on Drug Abuse (NIDA)-funded substance abuse treatment trial conducted at two adult correctional facilities in New Mexico. To be eligible, participants had to volunteer, meet DSM-IV (American Psychiatric Association, 1994) criteria for lifetime dependence on methamphetamine, heroin, or cocaine, and had to have used the drug within 3 months prior to their incarceration. Participants were given drug tests before each treatment session^2^, but due to ethical considerations, were given the opportunity to continue treatment and study participation regardless of test results. Exclusion criteria included: estimated full-scale IQ less than 70, less than a sixth grade reading level, current antipsychotic medication use, psychotic disorder diagnosis for self or a first-degree relative, or past or current central nervous system disease. Five participants were excluded due to motion during scanning or poor image quality (e.g., deformations due to dental work). The final sample consisted of 137 individuals (mean age = 34.03, *SD* = 8.18; 93 females; see Table 1 for race/ethnicity). Participants gave written informed consent and were compensated $1 per hour, commensurate with the rate of pay for labor inside correctional facilities. Study materials and procedures were approved by the UNM HSC Institutional Review Board.

**Table 1:** **Descriptive statistics (***N*** = **137**; males, females, and full sample) for demographic, cognitive, psychopathy, and substance use measures**.

**Variable**	**Males**	**Females**	***t*-statistic**	***df***	***p*-value**	**Full sample**
	**(*n* = 44)**	**(*n* = 93)**				**(*n* = 137)**
**DEMOGRAPHIC**						
Age	34.18 (8.76)	33.96 (7.93)	0.15	135	0.881	34.03 (8.18) 20–55
Handedness					0.220^c^	
Right	86.4%	79.6%				81.8%
Left	6.8%	10.8%				9.5%
Both/either	6.8%	3.2%				4.4%
Unavailable	0.0%	6.4%				4.4%
Ethnicity					0.266^c^	
Hispanic/latino	54.5%	65.6%				62.0%
Not hispanic/latino	45.4%	32.2%				36.5%
Unavailable	0.0%	2.2%				1.4%
Race					**<0.001^c^**	
White	54.5%	48.4%				50.4%
American Indian or Alaska native	2.3%	6.4%				5.1%
Asian	0.0%	1.1%				0.7%
Black or African American	9.1%	4.3%				5.8%
Other	2.3%	28.0%				19.7%
Did not wish to provide or unavailable	31.8%	11.8%				18.2%
**IQ^a^**	98.00 (13.32)	94.77 (8.05)	1.48	58.5	0.143	95.82 (10.12)
	74–120	74–117				74–120
**PSYCHOPATHY (PCL-R)**						
Total	22.62 (7.42)	19.00 (5.09)	2.92	62.8	**0.005**	20.17 (6.15)
	7.0–36.8	4.4–28.4				4.4–36.8
	skew = −0.16	skew = −0.43				skew = 0.06
Factor 1	6.81 (3.41)	4.42 (2.40)	4.19	63.7	**<0.001**	5.19 (2.97)
	0.0–14.0	0.0–12.0			**<0.001**	0.0–14.0
	skew = 0.16	skew = 0.63				skew = 0.69
Factor 2^b^	13.38 (3.71)	12.66 (3.03)	1.20	134	0.230	12.89 (3.27)
	4.0–20.0	4.4–19.0				4.0–20.0
	skew = −0.50	skew = −0.38				skew = −0.37
**SUBSTANCE USE**						
Drug of choice					0.496^c^	
Methamphetamine	43.2%	49.5%				47.4%
Cocaine	36.4%	37.6%				37.2%
Heroin	20.5%	12.9%				15.3%
Number of dependence diagnoses	2.64 (1.40)	2.69 (1.22)	−0.22	135	0.826	2.67 (1.28)
Years of regular use^d^	44.58 (22.65)	39.59 (21.76)	1.22	131	0.224	41.20 (22.09)

PCL-R, Psychopathy Checklist-Revised (Hare, 2003). df, degrees of freedom. Data are presented as means with standard deviations in parentheses, unless otherwise noted. Ranges and skewness values are also given for relevant variables. Levels of significance are for two-tailed independent samples t-tests, unless otherwise noted. Significant p-values are denoted with bold type.

a One female was missing an IQ estimate.

b One female had too many Factor 2 items omitted to calculate a score.

c Fisher's exact test p-value.

d Four participants were missing the years of regular use measure (three female).

### Assessments

#### Psychopathy

Psychopathy was assessed using the Hare Psychopathy Checklist-Revised (Hare, 2003) (PCL-R). Trained researchers reviewed institutional records and conducted semi-structured interviews regarding participants' psychosocial histories and interpersonal and emotional skills. Individuals are rated on a 3-point scale for 20 items, with scores ranging from 0 to 40. Traditional factor analyses of the PCL-R have revealed a two-factor structure (Harpur et al., 1989; Hare, 2003), although other models have been developed (Cooke and Michie, 2001), including a four facet model (Hare, 2003). Factor 1 (potential range: 0–16) comprises interpersonal (e.g., grandiosity, deceitfulness; Facet 1) and affective traits (e.g., lack of empathy, shallow affect; Facet 2), and Factor 2 (potential range: 0–20) comprises lifestyle behavioral (e.g., impulsivity, stimulation-seeking; Facet 3) and antisocial traits (e.g., poor behavioral controls, early behavioral problems; Facet 4). Interviews were videotaped to conduct reliability assessments. Double ratings were conducted on 16.8% of the sample, selected randomly. The intraclass correlation coefficient (ICC_1_one-way random effects model) was 0.83.

#### Substance use

Substance use was assessed in two ways:

1. *Number of Substance Dependence Diagnoses*. Trained researchers interviewed participants using the Structured Clinical Interview for DSM-IV disorders (First et al., 1997) (SCID) to assess lifetime dependence according to DSM-IV criteria. The total number of substances including alcohol for which an individual met lifetime dependence criteria was calculated (scale: 0–8).
2. *Years of Regular Substance Use*. A modified version of the Addiction Severity Index (McLellan et al., 1992) (ASI) was used to calculate the cumulative years of regular use (i.e., three or more times per week) for all substances (alcohol, heroin, cocaine, methamphetamine, cannabis, hallucinogens, and inhalants) combined. The ASI is a brief interview that asks details about the duration, frequency, and amount of use of multiple types of drugs.

#### Other measures

Vocabulary and Matrix Reasoning subtests of the Wechsler Adult Intelligence Scale (Wechsler, 1997) were used to estimate the full-scale IQ (Ryan et al., 1999). The Wide Range Achievement Test Word Reading subtest (Wilkinson, 1993) (WRAT-3) was used to assess reading level. The SCID was used to assess past and current Axis I disorders. Individuals self-reported their primary drug of choice (i.e., methamphetamine, cocaine, or heroin). See Table 1 for descriptive statistics on demographic and study variables.

### Stimuli and task

Imaging data were collected prior to the participants' initiating the study's 12-week treatment program. Two types of pictures (32 drug-related and 32 neutral) were selected from the popular media. Drug-related pictures depicted drugs or drug paraphernalia related to cocaine, heroin, and/or methamphetamine (e.g., white powder with a razor blade, a hand holding a syringe, a pipe). Neutral pictures depicted non-drug objects and scenes (e.g., white fluffy clouds, folded hands, a pen). Participants were instructed that they would see a series of pictures presented one at a time for 6 s. For each picture they were told to determine if anything in the picture gave them a craving feeling or desire to use drugs. Then they were instructed to rate their intensity of drug craving (in the form of a growing red bar) on a scale from 1 (no craving) to 5 (extreme craving) based on their immediate level of desire, not how they think they should feel or would hope to feel. After the rating screen, a black screen with a white fixation cross was presented for 4 s. Twenty null fixation trials the same duration as picture trials (i.e., picture + rating + fixation = 14 s) were interspersed randomly. Each participant completed two runs of 52 trials (16 drug-cues, 16 neutral, and 20 null fixation stimuli per run).

### MRI data acquisition and statistical analysis

Participants were scanned on the Mind Research Network 1.5T Siemens Avanto mobile MRI, stationed at the correctional facilities, using an EPI gradient-echo pulse sequence (TR 2000 ms, TE 39 ms, flip angle 75°, FOV 24 × 24 cm, 64 × 64 matrix, 3.8 × 3.8 mm in-plane resolution, 4 mm slice thickness with 1 mm gap, 27 slices).

Data were preprocessed and analyzed using Statistical Parametric Mapping software (SPM5; http://www.fil.ion.ucl.ac.uk/spm). The ArtRepair Toolbox (Mazaika et al., 2009) was used to detect and remove severe image artifacts. ArtRepair-detected artifactual images were replaced and a regressor was created to remove the effects of the outliers in the statistical analyses. Following ArtRepair each run was realigned using INRIAlign, a motion-correction algorithm that is unbiased by local signal changes (Freire et al., 2002). The six realignment parameters (three translations and three rotations) and second-order movement parameters were entered as covariates in the statistical models below in order to remove variance due to movement. Realigned images were spatially normalized to the Montreal Neurological Institute (MNI) template and smoothed with an 8 mm full-width at half-maximum (FWHM) Gaussian smoothing kernel. Low frequency noise was removed using a high pass filter (cutoff: 1/128 s). Pictures (drug-related and neutral), ratings, and null fixation trials were modeled separately. Pictures were modeled with the standard SPM hemodynamic response function. For each participant, images that represented the hemodynamic response associated with viewing drug-related vs. neutral pictures were computed.

One-sample *t*-tests in SPM5 were used to detect differences in viewing drug-related pictures vs. neutral pictures (i.e., main effects), and multiple regression analyses were used to evaluate the relationship between psychopathy and the neural associates of drug craving. PCL-R Total score was the predictor of most interest. PCL-R factors and facets were also examined to observe the unique variance accounted for by each factor or facet.

In addition to the primary regression analyses (one for PCL-R Total score, one for the factors, and one for the facets), three supplementary analyses were performed to evaluate the robustness of the effect of PCL-R Total score on the hemodynamic response to drug-related stimuli after including potential covariates (i.e., participant sex, age, number of substance dependence diagnoses). Additionally, one supplementary analysis was performed to ensure that the effects of interest were not due to the fact that some subjects had a positive (*n* = 11) or invalid/missing (*n* = 11) urinalysis just prior to the first treatment session. All whole-brain analyses were thresholded at *p* < 0.005 uncorrected for multiple comparisons with an extent of 10 voxels.

Regions of interest (ROIs) from the previously-cited craving and psychopathy literatures were evaluated using anatomical small volume correction analyses with PCL-R Total score as the predictor of interest and no covariates. These regions were: anterior cingulate, posterior cingulate, medial and lateral orbitofrontal cortex, amygdala, insula, hippocampus, thalamus, and parts of the basal ganglia (caudate, putamen, globus pallidus, and nucleus accumbens). ROIs were created using WFU PickAtlas (Maldjian et al., 2003) and the automated anatomical labeling (AAL) atlas (Tzourio-Mazoyer et al., 2002) in SPM5. An ROI was created for each region in each hemisphere, with the exception of structures that fall on the midline of the brain (i.e., anterior cingulate, posterior cingulate, and medial orbitofrontal cortex).

## Results

### Correlations

PCL-R Total score was significantly positively related to the two substance use variables: number of dependence diagnoses, *r* = 0.28, *p* = 0.001 and years of regular use, *r* = 0.28, *p* = 0.001. Factor 1, when controlling for Factor 2, was significantly positively related to years of regular use, *r* = 0.21, *p* = 0.018. Factor 2, when controlling for Factor 1, was significantly positively related to the number of dependence diagnoses, *r* = 0.36, *p* < 0.001. See Tables 2, 3 for more.

**Table 2:** **Pearson product-moment correlations (zero-order) between PCL-R Total scores and assessment variables**.

	**PCL-R total**	**Age**	**IQ**	**Number of dependence diagnoses**	**Years of regular use**	**Neutral ratings**	**Craving ratings**
Age	−0.08						
IQ	0.14	0.05					
Number of dependence diagnoses	0.28^***^	0.11	0.08				
Years of regular use	0.28^***^	0.46^****^	0.02	0.35^****^			
Neutral ratings	0.07	−0.07	0.05	0.14	0.10		
Craving ratings	0.16	−0.26^**^	0.01	0.15	0.11	0.32^****^	
Craving—neutral (difference scores)	0.13	−0.24^**^	−0.02	0.08	0.06	−0.19^*^	0.87^****^

* Correlation is significant at the 0.05 level;

** Correlation is significant at the 0.01 level;

*** Correlation is significant at the 0.005 level;

**** Correlation is significant at the 0.001 level; All correlations were two-tailed tests.

**Table 3:** **Pearson product-moment correlations (partial) between PCL-R factors and assessment variables**.

	**Factor 1, controlling for**	**Factor 2, controlling for**
	**Factor 2**	**Factor 1**
Age	0.13	−0.23^*^
IQ	−0.03	0.15
Number of dependence diagnoses	−0.08	0.36^****^
Years of regular use	0.21^*^	0.06
Neutral ratings	0.01	0.03
Craving ratings	0.03	0.13
Craving— neutral (difference scores)	0.03	0.12

* Correlation is significant at the.05 level;

**** Correlation is significant at the.001 level. All correlations were two-tailed tests.

### Behavioral craving ratings

Participants rated drug-related pictures (*M* = 2.58, *SD* = 0.96) as eliciting significantly more craving than neutral pictures [*M* = 1.51, *SD* = 0.48; main effect of picture type: *F*_(1, 135)_ = 174.17, *p* < 0.001]. There were no differences in craving by sex [main effect of sex: *F*_(1, 135)_ = 1.31, *p* = 0.255] and no interaction, *F*_(1, 135)_ = 1.82, *p* = 0.181. Additionally, there were no differences in craving ratings grouped by individuals' primary drug of choice, *F*_(2, 134)_ = 0.74, *p* = 0.479.

Craving ratings were not significantly related to PCL-R scores, Total: *r* = 0.16, *p* = 0.060; Factor 1: *r* = 0.03, *p* = 0.706 (controlling for Factor 2); Factor 2: *r* = 0.13, *p* = 0.142 (controlling for Factor 1). Craving ratings were also not significantly related to the number of dependence diagnoses, *r* = 0.15, *p* = 0.090 or years of regular use, *r* = 0.11, *p* = 0.195.

### Brain imaging

#### Main effect of picture type

**Figure 1:**
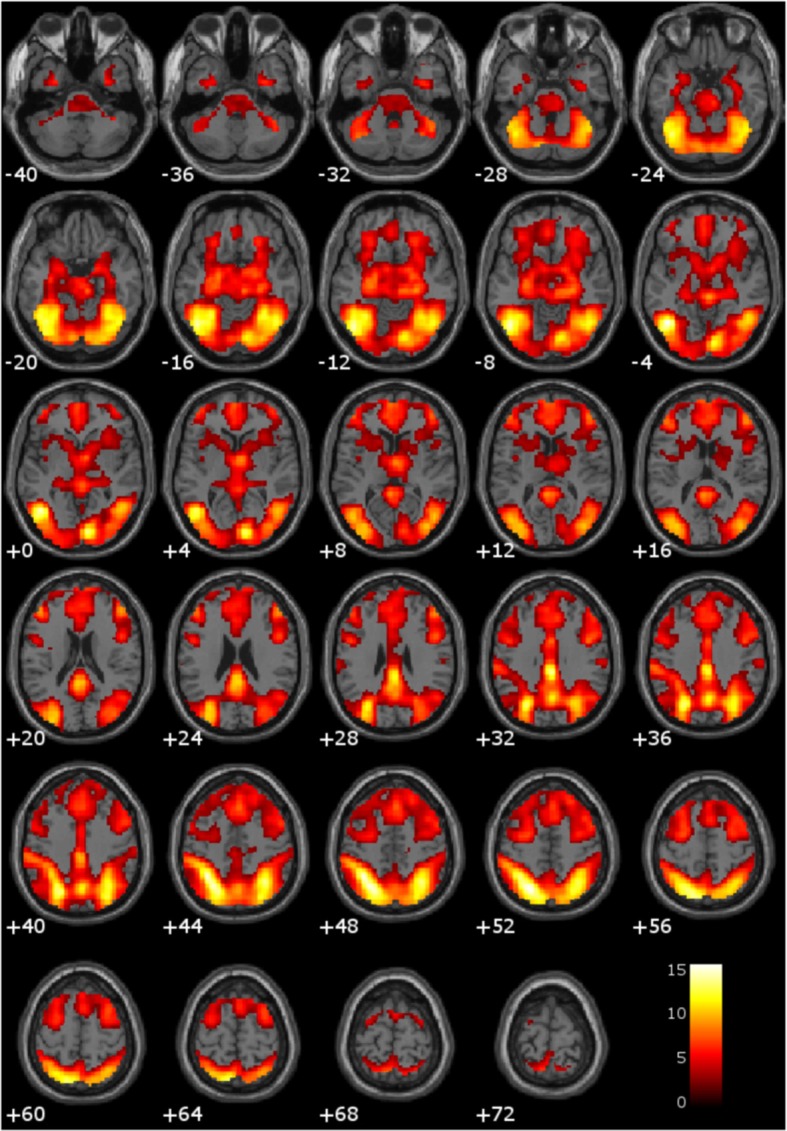
**Main effect of viewing drug-related pictures vs. neutral pictures.** These regions are significant in the whole brain at *p* < 0.005 with a 10 voxel extent. Numeric values indicate the MNI *z*-coordinate of the slice, and the color bar represents *t*-values.

Consistent with previous literature, the comparison of hemodynamic activity associated with viewing drug-related pictures relative to neutral pictures showed engagement of anterior cingulate, bilateral anterior/mid insula, bilateral hippocampus, bilateral amygdala, posterior cingulate, bilateral striatum (i.e., caudate, putamen, nucleus accumbens), and bilateral thalamus (Figure 1).

#### Modulating effects of psychopathy

**Figure 2:**
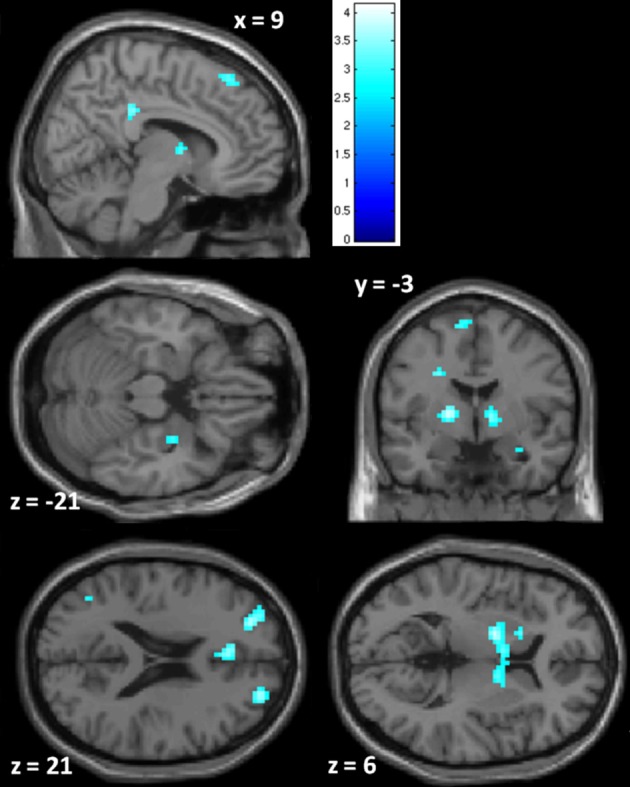
**Negative associations between PCL-R Total scores and hemodynamic activity for viewing drug-related pictures vs. neutral pictures.** These regions are significant in the whole brain at *p* < 0.005 with a 10 voxel extent. Numeric values indicate the MNI *z*-coordinate of the slice, and the color bar represents *t*-values.

***PCL-R Total scores (whole-brain analyses)***. As predicted, a significant negative association was found between PCL-R Total scores and hemodynamic activity to drug-related cues in areas such as the superior and middle frontal gyri, lentiform nucleus (lateral globus pallidus and putamen), caudate head, anterior cingulate, precentral gyrus, and inferior parietal lobule (for full results see Figure 2; Tables 4, A1). There was also one cluster showing a positive association between PCL-R Total scores and the neural associates of drug craving in the lingual gyrus (BA 18; *x* = 18, *y* = −75, *z* = −3; *k* = 20; *t* = 3.55, *p* < 0.001).

**Table 4:** **Negative associations between PCL-R Total scores and hemodynamic activity for viewing drug-related pictures vs. neutral pictures**.

**Label**	**BA**	***x***	***y***	***z***	**Hemi**	***t*-value**	***p*-value (unc.)**	**Cluster size^a^**
Superior frontal gyrus	8	15	30	57	R	4.14	<0.001	283
Middle frontal gyrus	8	−39	21	48	L	3.05	0.001	69
Inferior frontal gyrus	45	45	30	0	R	3.19	0.001	14
Anterior cingulate	32	−15	39	12	L	3.18	0.001	112 (a)
Anterior cingulate	24	−6	33	21	L	3.43	<0.001	112 (a)
Cingulate gyrus (Mid)	23	−6	−12	36	L	3.85	<0.001	78 (b)
Posterior cingulate	23	9	−33	33	R	3.35	0.001	78 (b)
Precuneus	31	18	−48	39	R	3.27	0.001	21
Lateral globus pallidus	−	15	0	0	R	3.50	<0.001	173 (c)
Lateral globus pallidus	−	−18	−3	6	L	3.81	<0.001	173 (c)

These areas are significant across four separate models: PCL-R Total score only, PCL-R Total score and participant sex, PCL-R Total score and age, PCL-R Total score and number of substance dependence diagnoses. BA, Brodmann Area; Hemi, Hemisphere. Coordinates are in MNI space. Areas are significant at uncorrected p < 0.005, extent threshold = 10 voxels.

a Cluster sizes are given for the PCL-R Total score only model. Letters in parentheses indicate a single cluster with multiple peak coordinates.

Three supplementary analyses were performed to control for the effects of potential covariates (participant sex, age, number of substance dependence diagnoses) on hemodynamic activity associated with viewing drug-related pictures. Each covariate was added as a nuisance variable to separate models that compared drug-related pictures to neutral pictures, with PCL-R Total score as the main predictor. Areas that were significant in all four models can be found in Table 4; other regions are listed in Table A1. A final supplementary analysis was performed to exclude the potential effect of active drug use (i.e., those with a positive or invalid/missing drug urinalysis were not included). There were negative associations with PCL-R Total scores in superior and middle frontal areas, the anterior, mid, and posterior cingulate, middle temporal areas, and globus pallidus, caudate, and putamen (Table A2). A test of the proportion of negative, positive, and invalid/missing urinalyses in each PCL-R category (where PCL-R ≤20 = low, 20–30 = middle, and ≥30 = high) was not significant (*p* = 0.085, Fisher's exact test).

***PCL-R factor and facet scores (whole-brain analyses)***. The negative effect of psychopathy on the hemodynamic response to drug-related stimuli was most strongly correlated with the social deviance factor, Factor 2 (Table A3). In addition to the regions identified in the analysis with PCL-R Total scores, there was significantly reduced engagement of the left amygdala with higher Factor 2 scores. There were no negative associations between Factor 1 (controlling for Factor 2) and viewing drug cues.

There were significant positive associations between neural activity for viewing drug cues and Factor 1 (controlling for Factor 2) in the cerebellum, precentral gyrus, lateral globus pallidus, middle occipital gyrus, and insula. There were no positive associations between Factor 2 (controlling for Factor 1) and neural activity for viewing drug cues.

Relationships between the hemodynamic response to drug-related stimuli and the PCL-R facets are presented in the Appendix text (Table A4).

***Region of interest analyses***. Using small volume correction with ROIs related to psychopathy and/or drug craving, the right putamen and left and right globus pallidus were significantly negatively related to PCL-R Total scores. Additionally, there was trend-level significance in the anterior cingulate, posterior cingulate, right amygdala, and left thalamus, caudate, putamen, and nucleus accumbens (Table 5).

**Table 5:** **Small volume correction associations between PCL-R Total scores and hemodynamic activity for viewing drug-related pictures vs. neutral pictures in anatomical regions of interest (ROIs)**.

**Region**	***x***	***y***	***z***	**FWE *p*-value**	**FDR *p*-value**	***t*-value**	**Cluster size**
*Anterior cingulate*	−6	*33*	*21*	*0.084*	*0.080*	*3.43*	*832*
*Posterior cingulate*	*6*	−*36*	*30*	*0.096*	*0.083*	*3.00*	*244*
*R Amygdala*	*30*	−*3*	−*21*	*0.069*	*0.187*	*2.69*	*75*
*L Thalamus*	−*15*	−*6*	*6*	*0.083*	*0.181*	*3.07*	*351*
*L Caudate*	−*6*	*3*	*6*	*0.061*	*0.073*	*3.21*	*292*
**R Putamen**	**24**	**6**	**−3**	**0.084**	**0.046**	**3.09**	**312**
*L Putamen*	−*18*	*0*	*9*	*0.099*	*0.089*	*3.01*	*293*
*L Nucleus accumbens*	−*6*	*9*	−*9*	*0.081*	*0.064*	*2.15*	*17*
**R Globus pallidus**	**15**	**0**	**0**	**0.009**	**0.009**	**3.50**	**82**
**L Globus pallidus**	**−18**	**−3**	**6**	**0.004**	**0.005**	**3.81**	**86**

R, right; L, left; FWE, family-wise error. FDR, false discovery rate. Coordinates are in MNI space. Entries in bold face type are significant at a corrected threshold of p < 0.05; entries in italics are significant at a corrected threshold of p < 0.10 (i.e., trend level). ROIs that did not produce significant results were: lateral and medial orbitofrontal cortex, left amygdala, left and right hippocampus, left and right insula, right thalamus, right caudate, and right nucleus accumbens.

## Discussion

This study tested the hypothesis that psychopathic traits would negatively modulate the hemodynamic response to drug-related stimuli for three types of drugs and different routes of administration in male and female substance-dependent inmates. Consistent with hypotheses, psychopathy scores were negatively associated with brain engagement to drug-related cues in several limbic and paralimbic areas, including the anterior cingulate, posterior cingulate, striatum, and amygdala.

The anterior cingulate is involved in cognitive control, conflict monitoring, and error detection (Kiehl et al., 2000; Kerns et al., 2004). Previous studies have reported less anterior cingulate activity in psychopathy during the performance of various tasks, including aversive conditioning (Veit et al., 2002; Birbaumer et al., 2005), negative picture viewing (Muller et al., 2003) and affective word recognition (Kiehl et al., 2001). Though cognitive control during a cue-elicited craving task in psychopathy has not been investigated previously, one possibility is that in the present study, high psychopathy scorers experienced reduced conflict in response to drug cues due to reduced craving. In other words, unlike low-scorers, those scoring high on psychopathy did not perceive drug cues to cause cognitive conflict. Alternatively, it may be that gray matter abnormalities lead to less hemodynamic activity in the anterior cingulate cortex. At least two studies have associated psychopathy with reduced gray matter in the anterior cingulate in adult males (Boccardi et al., 2011; Ly et al., 2012). The specificity of hemodynamic abnormalities in the anterior cingulate remains an open question.

The amygdala is responsible for placing emotional significance on associations between relevant stimuli (Everitt et al., 2003). Drug craving involves engagement of the amygdala, in part because of the acquired emotional significance of drug cues (Everitt and Wolf, 2002). The negative correlation between psychopathy scores and amygdala reactivity to drug cues is consistent with the well-documented dysfunction of this region in psychopathy during aversive conditioning (Birbaumer et al., 2005), emotional word recognition (Kiehl et al., 2001), and moral decision-making (Harenski et al., 2010). Blair (2008) and others (Kiehl, 2006) have identified amygdala dysfunction as being central to the development of psychopathy.

Engagement of parts of the basal ganglia and striatum while viewing drug-related cues was negatively related to psychopathy scores. These brain areas are involved in drive-related behavior and take part in making associations between stimuli and rewards. The ventral striatum (i.e., nucleus accumbens), including the region where the putamen and head of the caudate meet, receives projections from the amygdala (Nolte, 2002). One possibility here is that the amygdala is not signaling the emotional significance of rewarding stimuli to the same extent in high psychopathy-scorers compared to low-scorers, and thus the regions in the basal ganglia and striatum are under-reactive to drug-related cues. Other work has suggested instead a heightened sensitivity to reward with higher psychopathic traits (Buckholtz et al., 2010; Bjork et al., 2012), but these studies differed crucially from the present work in that both utilized community volunteers without histories of substance use. Future work involving connectivity analyses could address these alternative interpretations.

There was also a negative relationship between psychopathy scores and hemodynamic activity to drug cues in the posterior cingulate. Prior research demonstrated that smokers trying to resist cue-elicited craving showed engagement of the posterior cingulate (Brody et al., 2007). Thus, PCL-R low-scorers may have engaged the posterior cingulate in order to suppress their craving response. In contrast, high-scorers had less posterior cingulate engagement because there was less craving to inhibit. Another explanation for attenuated posterior cingulate engagement to drug cues may be that individuals higher in psychopathy have less gray matter volume in the posterior cingulate overall (Ermer et al., 2012).

Whereas PCL-R Total score showed the strongest negative correlations with drug-related hemodynamic activity, the social deviance component of the PCL-R (Factor 2) and the lifestyle and antisocial facets (Facet 3 and 4) appeared to be more strongly correlated with drug-related hemodynamic activity than were the affective and interpersonal components of psychopathy (i.e., Factor 1 and Facets 1 and 2). Factor 2 (and Facet 4 in particular) has sometimes been criticized for capturing only the behavioral consequences of the Factor 1 personality traits that some argue are more central to the concept of psychopathy (Cooke and Michie, 2001), but it is important to note that effects observed in the present study cannot be explained by general social deviance *per se*. The lifestyle facet of psychopathy, Facet 3, includes items related to chronic instability and impulsivity. Impulsivity, particularly the shortsightedness aspect, has always been important to the construct of psychopathy (Hare, 2003), and at least one study found significant correlations between components of impulsivity (e.g., nonplanning impulsivity) and PCL-R Factor 2 scores (Snowden and Gray, 2011).

With respect to the negative relationship between drug-related hemodynamic activity and Facet 4 of psychopathy it is important to keep a few ideas in mind. Whereas Facet 4 was originally labeled the *antisocial* facet of psychopathy, it includes the PCL-R items that directly assess severe behavioral problems that arise early in life [i.e., early behavioral problems before age 12 (PCL-R item 10)], persist through adolescence [i.e., severe juvenile delinquency (PCL-R Item 18)], and continue into adulthood [i.e., many antisocial behaviors (PCL-R Item 20); failure during conditional release (PCL-R Item 19)]. Thus, Facet 4 is the only facet of psychopathy that represents the *developmental course* of the condition.

One possible explanation for the present neuroimaging results comes from the incentive sensitization theory of addiction (Robinson and Berridge, 1993), which describes three distinct processes involved in the development of reward learning: (1) *pleasure* (i.e., “liking”), (2) *associative learning* of the link between targets and their hedonic value, and (3) *attribution of incentive salience* to those targets (i.e., “wanting/craving”). Distinct neural systems mediate these processes, allowing for differential expression and behavior. In individuals who are addicted to drugs, it is thought that increased incentive salience (process #3) mediates compulsive drug-seeking and drug-taking (Robinson and Berridge, 1993). The self-reported subjective pleasure associated with drug-taking (process #1) does not become sensitized. This distinction is supported by individuals' reports that they *like* drugs less and less the more they take them, but they *want* the drugs more and more (i.e., craving).

Evidence primarily from animal studies indicates that incentive salience attribution is mediated by the mesocorticolimbic dopamine system (Berridge, 2009), involving the nucleus accumbens, amygdala, hippocampus, and medial prefrontal cortex, including the anterior cingulate. Keeping in mind the developmental aspect of Facet 4, perhaps psychopaths' incentive salience (i.e., “wanting” system) fails to be sensitized due to abnormal neurodevelopment in critical areas, while the motivating factor is positive reinforcement for drugs' pleasurable effects. Thus, relative to non-psychopaths, psychopaths may have different motivations for using drugs, where craving does not act as a potent motivator (see also Berridge, 2012). Our finding that higher psychopathy scores are associated with reduced hemodynamic activity in response to drug cues is consistent with the explanation that psychopaths experience reduced or absent incentive salience sensitization, and therefore reduced craving when access to drugs is externally limited (e.g., during incarceration). Future work should attempt to clarify how drug craving is related to the different components of psychopathy.

Because craving has been shown to be a predictor of relapse (Weiss, 2005), future studies should consider examining the role of psychopathic traits in drug treatment efficacy. Psychopathic traits appear to be important variables to consider when designing treatment strategies, as these data demonstrate differences in response to drug cues with different levels of psychopathic traits, and indicate the possibility of differences in the time course of—and reasons for—drug relapse. In terms of generalizability of findings, the sample comprised mostly polydrug users (mean number of substance dependence diagnoses = 2.7), which is more common than single drug use among treatment seekers (SAMHSA, 2011). The present sample is thus representative of the typical drug user.

## Conclusions

Numerous functional imaging studies have associated psychopathy with dysfunction in limbic and paralimbic regions, many of which have also been implicated in drug craving. This study found a negative association between psychopathy scores and engagement of craving-related areas during the viewing of drug cues. Abnormal activity in limbic and paralimbic areas in psychopathy has been demonstrated in multiple domains, including moral decision-making (Harenski et al., 2010), fear conditioning (Birbaumer et al., 2005), emotional memory (Kiehl et al., 2001), and now responses to drug cues. It will be important to follow up on the present line of work in order to develop more effective and efficient drug abuse treatments by considering an individual's level of psychopathy when determining the most effective treatment strategy. This work has the potential to reduce the extreme burden—both financial and otherwise—of drug use disorders.

## Author contributions

Kent A. Kiehl conceived of the study. Lora M. Cope and Justin L. Jobelius collected the data. Lora M. Cope, Justin L. Jobelius, and Prashanth K. Nyalakanti analyzed the data, with assistance from Gina M. Vincent, Kent A. Kiehl, and Vince D. Calhoun. Lora M. Cope, Justin L. Jobelius, Gina M. Vincent, and Kent A. Kiehl interpreted the data and wrote the manuscript, with input from Vince D. Calhoun and Prashanth K. Nyalakanti.

### Conflict of interest statement

The authors declare that the research was conducted in the absence of any commercial or financial relationships that could be construed as a potential conflict of interest.

## Footnotes

^1^In other words, greater blood oxygenation level dependent response, regional cerebral blood flow, or glucose metabolism to drug cues vs. neutral cues.

^2^Substance abuse treatment began within 1 week of the initial fMRI scan, barring personal or institutional circumstances (e.g., participant sickness, institutional lock-down) that precluded the participant from keeping the appointment.

## Appendix

Psychopathic Traits Modulate Brain Responses to Drug Cues in Incarcerated Offenders.

**Table A1:** **Negative associations between PCL-R Total scores and hemodynamic activity for viewing drug-related pictures vs. neutral pictures in areas that were not significant in all four models (PCL-R Total score only, PCL-R Total score and participant sex, PCL-R Total score and age, PCL-R Total score and number of substance dependence diagnoses) (cf. Table 4)**.

**Label**	**BA**	***x***	***y***	***z***	**Hemi**	***t*-value**	***p*-value (unc.)**	**Cluster size^a^**	**In other models?**
Superior frontal gyrus	6	−9	−3	66	L	2.97	0.002	10 (a)	^∧^
Middle frontal gyrus	46	−39	36	27	L	2.81	0.003	15	^#^^∧^
Middle frontal gyrus	9	30	42	42	R	3.94	<0.001	283	^#^^∧^
Inferior frontal gyrus	9	−57	9	30	L	2.99	0.002	16 (b)	^#^
Inferior frontal gyrus	9	−48	12	27	L	2.80	0.003	16 (b)	^*^
Precentral gyrus	9	−42	3	42	L	3.07	0.001	69	^#^^∧^
Precentral gyrus	6	45	−6	24	R	3.31	0.001	10 (c)	^∧^
Cingulate gyrus (mid)	24	3	−21	36	R	3.04	0.001	78	^#^^∧^
Inferior parietal lobule	40	−39	−60	42	L	3.27	0.001	24	^*^^#^
Inferior parietal lobule	40	−51	−42	36	L	3.01	0.002	46 (d)	^#^
Inferior parietal lobule	40	−48	−39	45	L	2.98	0.002	46 (d)	^#^^∧^
Superior temporal gyrus	39	48	−51	12	R	3.16	0.001	11	^@^
Middle temporal gyrus	–	54	−36	−6	R	2.70	0.004	13	%
Caudate (head)	–	−6	3	6	L	3.21	0.001	173	^*^
Putamen	–	−18	15	6	L	3.11	0.001	10 (e)	^#^^∧^

BA, Brodmann Area; Hemi, Hemisphere. Coordinates are in MNI space. Areas are significant at uncorrected p < 0.005, extent threshold = 10 voxels.

a Cluster sizes are given for the PCL-R Total score only model.Letters in parentheses distinguish different clusters of the same size or indicate a single cluster with multiple peak coordinates.

@ Present when including PCL-R Total score only.

* Present when including PCL-R Total score and participant sex.

# Present when including PCL-R Total score and age.

∧ Present when including PCL-R Total score and number of substance dependence diagnoses.

**Table A2:** **Negative associations between PCL-R Total scores and hemodynamic activity for viewing drug-related pictures vs. neutral pictures in participants with a valid, negative urinalysis at baseline screening (*n* = 115)**.

**Label**	**BA**	***x***	***y***	***z***	**Hemi**	***t*-value**	***p*-value (unc.)**	**Cluster size^a^**
Superior frontal gyrus	6	15	27	57	R	3.87	<0.001	55 (a)
	10	−24	48	18	L	3.37	0.001	85 (b)
	9	30	45	36	R	3.60	<0.001	74 (c)
Middle frontal gyrus	10	27	54	24	R	2.83	0.003	74 (c)
	8	39	30	48	R	2.88	0.002	74 (c)
	9	−42	33	30	L	3.38	0.001	44
	9	−36	12	36	L	3.64	<0.001	53 (d)
	9	−42	6	42	L	3.01	0.002	53 (d)
	8	21	33	48	R	2.81	0.003	55 (a)
	47	−42	33	−3	L	3.18	0.001	12 (e)
Precentral gyrus	4	30	−21	48	R	3.96	<0.001	16
Anterior cingulate	32	−15	39	12	L	3.60	<0.001	85 (b)
	32	−9	36	21	L	3.32	0.001	85 (b)
Cingulate gyrus (mid)	24	3	−21	36	R	3.74	<0.001	94 (f)
	23	−6	−12	36	L	3.71	<0.001	94 (f)
Posterior cingulate	23	9	−33	30	R	3.69	<0.001	94 (f)
Precuneus	31	3	−51	30	R	3.18	0.001	15
	31	18	−48	39	R	3.43	<0.001	21
Superior temporal Gyrus	39	48	−51	9	R	3.39	<0.001	17
Middle temporal Gyrus	22	48	−39	0	R	3.02	0.002	31 (g)
	20	54	−39	−9	R	3.35	0.001	31 (g)
	21	51	−18	−18	R	3.59	<0.001	37
	21	−63	−36	−12	L	3.51	<0.001	77 (h)
	21	−60	−24	−18	L	3.07	0.001	77 (h)
	20	−51	−33	−12	L	3.18	0.001	77 (h)
Supramarginal gyrus	40	−42	−48	36	L	2.83	0.003	19 (i)
Inferior parietal lobule	40	−39	−39	30	L	3.57	<0.001	19(i)
	40	−48	−39	45	L	3.02	0.002	12 (j)
	40	−39	−60	39	L	3.00	0.002	10
Lateral globus pallidus	–	15	0	0	R	3.25	0.001	36
Caudate (head)	–	−3	3	6	L	3.49	<0.001	45
Putamen	–	−21	−3	3	L	3.21	0.001	19

BA, Brodmann Area; Hemi, Hemisphere. Coordinates are in MNI space. Areas are significant at uncorrected p < 0.005, extent threshold = 10 voxels.

a Letters in parentheses distinguish different clusters of the same size or indicate a single cluster with multiple peak coordinates.

**Table A3:** **Associations between PCL-R factor scores and hemodynamic activity for viewing drug-related pictures vs. neutral pictures**.

		**Label**	**BA**	***x***	***y***	***z***	**Hemi**	***t*-value**	***p*-value (unc.)**	**Cluster size^a^**
Factor 1	Positive^*^	Precentral gyrus	6	39	−12	45	R	3.28	0.001	11
		Insula	13	−39	−9	3	L	3.09	0.001	13
		Middle occipital gyrus	18	15	−102	12	R	3.40	<0.001	12
		Lateral globus pallidus	–	−21	−12	−9	L	3.30	0.001	11
		Culmen (cerebellum)	–	18	−33	−21	R	3.35	0.001	30
			–	−24	−39	−24	L	3.25	0.001	14
Factor 2	Negative	Medial frontal gyrus	6	−12	−12	63	L	4.25	<0.001	100
		Superior frontal gyrus	6	15	−18	69	R	3.04	0.001	19 (a)
			6	15	24	57	R	3.30	0.001	108 (b)
			6	−12	21	54	L	2.87	0.002	19 (c)
			10	−18	48	18	L	2.72	0.004	116 (d)
			8	18	36	48	R	4.16	<0.001	108 (b)
			8	−6	27	57	L	3.06	0.001	19 (c)
			8	−18	15	45	L	2.75	0.003	19 (c)
		Middle frontal gyrus	8	−30	18	51	L	3.39	<0.001	23
			9	27	36	39	R	3.23	0.001	108 (b)
			9	−39	9	36	L	2.92	0.002	48 (e)
			46	−39	15	24	L	3.17	0.001	48 (e)
			6	−36	−3	48	L	3.17	0.001	17 (f)
		Inferior frontal gyrus	46	45	36	3	R	3.35	0.001	24 (t)
		Precentral gyrus	6	−27	−21	63	L	3.07	0.001	12 (g)
			6	−24	−21	72	L	2.72	0.004	12 (g)
		Postcentral gyrus	2	−60	−30	45	L	3.15	0.001	18 (h)
			3	15	−42	72	R	3.51	<0.001	15
		Parahippocampal gyrus	36	27	−27	−18	R	4.16	<0.001	28
		Hippocampus	–	27	−39	−6	R	4.04	<0.001	21
		Amygdala	–	−30	0	−27	L	3.62	<0.001	33
		Anterior cingulate	32	−18	39	12	L	3.83	<0.001	116 (d)
			32	−9	9	36	L	3.23	0.001	19 (i)
			24	−3	27	18	L	4.2	<0.001	116 (d)
		Cingulate gyrus (mid)	24	−6	−12	39	L	3.82	<0.001	80 (j)
			24	9	−12	36	R	3.28	0.001	80 (j)
		Posterior cingulate	31	12	−42	39	R	3.35	0.001	106 (k)
			31	3	−45	33	R	3.29	0.001	106 (k)
		Inferior parietal lobule	40	−39	−57	42	L	3.24	0.001	17 (l)
			40	42	−36	30	R	3.98	<0.001	22
		Superior temporal gyrus	39	54	−57	12	R	3.13	0.001	24 (m)
			22	45	0	−3	R	2.90	0.002	12 (n)
		Middle temporal gyrus	37	54	−63	3	R	2.86	0.002	24 (m)
		Transverse temporal gyrus	41	−39	−33	9	L	3.42	<0.001	16
		Insula	13	−39	−12	6	L	3.11	0.001	32 (o)
			13	−39	−3	0	L	2.81	0.003	32 (o)
		Middle occipital gyrus	18	−9	−93	12	L	2.96	0.002	19 (p)
		Cuneus	17	−3	−93	0	L	2.81	0.003	19 (p)
		Lateral globus pallidus	–	−21	−15	−9	L	3.67	<0.001	77 (q)
		Medial globus pallidus	–	−15	−3	0	L	3.32	0.001	77 (q)
		Caudate (tail)	–	−33	−15	−12	L	3.19	0.001	77 (q)
		Caudate (head)	–	12	12	6	R	2.93	0.002	28 (r)
		Caudate (body)	–	9	3	6	R	2.90	0.002	28 (r)
		Claustrum	–	36	−21	3	R	3.37	<0.001	32 (o)
			–	36	−3	3	R	2.82	0.003	12 (n)
			–	−36	−21	0	L	2.85	0.003	17 (s)

BA, Brodmann Area; Hemi, Hemisphere. Coordinates are in MNI space. Areas are significant at uncorrected p < 0.005, extent threshold = 10 voxels.

* Each coordinate is in a different cluster.

a Letters in parentheses distinguish different clusters of the same size or indicate a single cluster with multiple peak coordinates.

**Table A4:** **Associations between PCL-R facet scores and hemodynamic activity for viewing drug-related pictures vs. neutral pictures**.

		**Label**	**BA**	***x***	***y***	***z***	**Hemi**	***t*-value**	***p*-value (unc.)**	**Cluster size^a^**
Facet 1	Positive	Postcentral gyrus	2	51	−24	51	R	3.53	<0.001	75 (a)
		Precentral gyrus	4	36	−18	63	R	3.50	<0.001	75 (a)
		Superior temporal gyrus	22	60	0	−3	R	3.76	<0.001	17
			22	−57	−6	−3	L	3.09	0.001	16
		Insula	13	45	−9	9	R	3.42	<0.001	11 (b)
		Parahippocampal gyrus	36	−27	−39	−9	L	2.85	0.003	59 (c)
		Uncus	20	−33	−6	−33	L	3.35	0.001	23 (d)
			36	−24	−3	−36	L	2.76	0.003	23 (d)
		Putamen	–	−27	−15	−6	L	3.29	0.001	13
		Thalamus	–	21	−30	9	R	3.24	0.001	28
		Culmen (cerebellum)	–	−24	−36	−21	L	3.44	<0.001	59 (c)
			–	−30	−51	−30	L	2.73	0.004	59 (c)
			–	18	−33	−18	R	3.10	0.001	11 (e)
Facet 2	Positive	Cuneus	18	18	−78	21	R	3.40	<0.001	11
		Lateral globus pallidus	–	21	−6	−6	R	3.02	0.001	10
	Negative	Superior frontal gyrus	10	21	51	3	R	3.51	<0.001	26 (f)
		Anterior cingulate	32	21	42	3	R	3.35	0.001	26 (f)
		Superior temporal gyrus	38	45	12	−27	R	3.53	<0.001	24
			22	−57	−54	6	L	3.18	0.001	35 (g)
		Middle temporal gyrus	22	−54	−45	0	L	2.98	0.002	35 (g)
			38	−36	9	−42	L	3.15	0.001	14 (h)
			21	−42	0	−42	L	3.06	0.001	14 (h)
		Fusiform gyrus	20	36	−12	−27	R	3.17	0.001	14 (i)
		Thalamus	–	−21	−21	18	L	3.49	<0.001	38
Facet 3	Positive^*^	Medial frontal gyrus	9	12	39	36	R	3.15	0.001	12
		Middle frontal gyrus	11	−27	42	−9	L	3.06	0.001	15
		Inferior frontal gyrus	47	−45	21	−3	L	3.17	0.001	19
		Caudate (body)	–	6	12	15	R	3.97	<0.001	31
		Culmen (cerebellum)	–	−3	−33	−21	L	3.18	0.001	12
	Negative	Medial frontal gyrus	6	−12	−3	66	L	3.89	<0.001	302 (j)
			6	−6	−15	63	L	3.77	<0.001	302 (j)
		Middle frontal gyrus	6	−36	−3	48	L	3.10	0.001	15
		Postcentral gyrus	3	−30	−36	66	L	3.69	<0.001	302 (j)
			2	−36	−27	45	L	3.44	<0.001	112 (k)
			2	−42	−24	33	L	3.17	0.001	112 (k)
		Precentral gyrus	6	39	−9	45	R	3.63	<0.001	22
		Anterior cingulate	32	−15	33	18	L	3.27	0.001	11
			24	−9	6	39	L	2.94	0.002	50 (l)
		Cingulate gyrus	24	−9	−12	39	L	4.15	<0.001	50 (l)
		Posterior cingulate	31	12	−42	42	R	3.03	0.001	88 (m)
		Precuneus	7	0	−45	45	–	3.78	<0.001	88 (m)
			7	6	−69	51	R	3.01	0.002	13 (n)
		Fusiform gyrus	37	−45	−36	−12	L	4.01	<0.001	52 (o)
			20	−39	−30	−21	L	3.05	0.001	52 (o)
		Middle temporal gyrus	20	−54	−36	−12	L	3.58	<0.001	52 (o)
		Transverse temporal Gyrus	41	−33	−33	9	L	3.78	<0.001	38 (p)
		Superior temporal gyrus	41	−39	−30	3	L	3.43	<0.001	38 (p)
		Inferior parietal lobule	40	−60	−27	33	L	3.69	<0.001	112 (k)
		Cuneus	18	3	−84	21	R	3.39	<0.001	64
		Lingual gyrus	19	−15	−54	−6	L	3.24	0.001	23
				−9	−69	0	L	2.97	0.002	13 (q)
		Putamen	–	−33	−21	0	L	3.18	0.001	38 (p)
		Substantia nigra	–	−18	−18	−9	L	4.24	<0.001	25
		Culmen (cerebellum)	–	−24	−51	−18	L	3.07	0.001	10
Facet 4	Positive	Insula	13	−30	−12	21	L	3.45	<0.001	10
	Negative	Inferior frontal gyrus	47	−39	21	−21	L	4.51	<0.001	196 (q)
			47	−39	18	−6	L	4.05	<0.001	196 (q)
			47	−42	33	−6	L	3.58	<0.001	196 (q)
			47	48	36	3	R	4.04	<0.001	89 (r)
			45	57	27	15	R	3.92	<0.001	89 (r)
			47	42	18	−12	R	2.88	0.002	10 (s)
		Medial frontal gyrus	10	3	57	9	R	3.00	0.002	217 (t)
			8	−9	24	54	L	3.42	<0.001	19
			8	15	30	48	R	4.14	<0.001	75
			9	3	45	18	R	4.05	<0.001	217 (t)
		Middle frontal gyrus	9	−42	9	39	L	3.15	0.001	27 (u)
			9	30	18	36	R	3.65	<0.001	21 (v)
			46	−45	21	30	L	2.85	0.003	27 (u)
			46	−45	30	18	L	3.08	0.001	16
		Anterior cingulate	24	−3	27	27	L	3.66	<0.001	217 (t)
			32	−21	42	12	L	3.19	0.001	11
		Cingulate gyrus (mid)	24	9	−9	33	R	3.13	0.001	48 (w)
		Posterior cingulate	31	6	−45	30	R	3.55	<0.001	21 (x)
			29	−6	−51	9	L	3.34	0.001	20
			30	9	−51	12	R	3.27	0.001	21 (y)
		Amygdala	–	27	−3	−24	R	3.35	0.001	26 (z)
		Parahippocampal gyrus	35	24	−27	−21	R	3.90	<0.001	104 (aa)
			34	18	−9	−21	R	2.75	0.003	26 (z)
			27	21	−33	−6	R	3.01	0.002	24 (bb)
		Superior temporal gyrus	41	45	−36	9	R	3.19	0.001	10 (cc)
		Supramarginal gyrus	40	−51	−48	30	L	3.37	<0.001	25
		Cuneus	19	27	−84	27	R	3.21	0.001	13 (dd)
			19	27	−87	18	R	2.83	0.003	13 (dd)
		Caudate (body)	–	15	18	18	R	3.45	<0.001	40 (ee)
			–	12	12	9	R	3.03	0.001	40 (ee)
			–	15	−15	24	R	2.98	0.002	48 (w)
		Thalamus	–	0	−12	3	–	3.37	<0.001	34
			–	18	−27	3	R	2.83	0.003	24 (bb)
		Claustrum	–	−33	−6	3	L	3.41	<0.001	15 (ff)
		Culmen (cerebellum)	–	12	−33	−18	R	3.85	<0.001	104 (aa)

BA, Brodmann Area; Hemi, Hemisphere. Coordinates are in MNI space. Areas are significant at uncorrected p < 0.005, extent threshold = 10 voxels.

* Each coordinate is in a different cluster.

a Letters in parentheses distinguish different clusters of the same size or indicate a single cluster with multiple peak coordinates. Coordinates listed under different facets/directions are in different clusters.
